# 

**Published:** 1893-01-28

**Authors:** 


### CONTENTS.

**The Progress and Policy of The Hospitals Association** 275

**Hypnotism and Drunkenness** - - - - - - - - 276

**Medical History from the Earliest Times.** By E. T. WITHINGTON, M.A., M.B.Oxon. XL.—The Close of the Middle Ages - - - - - - - - - - - - 277

**Contemporary Theory and Research.**—Hypnotism for the Insane—Dermographism—No Quarantine - - - - - - 278

**Magic Cures** II. - - - - - - - - - - 279

**Everybody's Page** - - - - - - - - - 280

**Annotations.**—The "Bromide Habit"—Soft v. Hard Water—Honest Anti Vaccinators - - - - - - - - - 281

**Notes and News** - - - - - - - - - - 282

**Scraps and Gleanings** - - - - - - - - - 290

**The World's Fair**—The International Congress of Charities, Correction, and Philanthropy, June 1[illegible]—18, 1893. - - - 290

**The Hospital Clinic—**

ST. MARY'S HOSPITAL.—Treatment of Dilatation of the Stomach 283

MANCHESTER AND PENDLEBURY CHILDREN'S HOSPITAL.—The Treatment of Hip Disease - - - - - - - 284

BRISTOL CHILDREN'S HOSPITAL.—The Treatment of Hip Joint Disease - - - - - - - - - - - - 285

BIRMINGHAM HOSPITALS.—The Treatment of Pott's Fracture - 285

**New Drugs, Appliances, and Things Medical.**—An Admirable Hot-Water Bottle - - - - - - - - 286

**The Institutional Workshop—**

PRACTICAL DEPARTMENTS.—Cooking by Gas. IV. - - - 287

HOSPITAL ADMINISTRATION.—Hospitals and Asylums of the World - - - - - - - - - - - - 287

A CENTRAL HOSPITALS BOARD FOR LONDON - - - - - 288

**The Student of Medicine.**

### EXTRA SUPPLEMENT.—THE NURSING MIRROR.

**News from the Nursing World.**—Wanted, Good Nurses—Enthusiasm at Darlington—House of Recovery—Valued Nurses—An Association of French Ladies—The International Nursing Congress, Chicago—Airings for Young Patients — American Addresses - Institute Nurses in Australia—Short Items—A Disabled Nurse - - - - - cxxvii

**The Development of Children by Gymnastics.** VI.—Swedish Drill - - - - - - - - - - - cxxix

**Military Hospitals.** As Seen by a Nurse's Eyes - - - cxxx

**Bedsores** - - - - - - - - - - - - cxxxi

**Novelties for Nurses** - - - - - - - - - cxxxi

**Presentations** - - - - - - - - - - - cxxxi

**Everybody's Opinion.**—Holiday Homes—The Late Mrs. Wardroper — Girl Nurses and Mrs. Wardroper — "The Hospital" Endowed Bed—A Disabled Nurse - - - cxxxii

**International Nursing Congress, Chicago** - - - cxxxiii

**Appointments—Minor Appointments** - - - - cxxxiii

**Royal British Nurses' Association—Where to Go** - cxxxiii

**A Warning to Nurses** - - - - - - - - cxxxiv

**Notes and Queries—Wants and Workers** - - - cxxxiv

